# Ag/ZnO Thin Film Nanocomposite Membrane Prepared by Laser-Assisted Method for Catalytic Degradation of 4-Nitrophenol

**DOI:** 10.3390/membranes12080732

**Published:** 2022-07-24

**Authors:** Tahani A. Alrebdi, Hoda A. Ahmed, Fatemah H. Alkallas, Rami Adel Pashameah, Salhah H. Alrefaee, Emaan Alsubhe, Ayman M. Mostafa, Eman A. Mwafy

**Affiliations:** 1 Department of Physics, College of Science, Princess Nourah bint Abdulrahman University, P.O. Box 84428, Riyadh 11671, Saudi Arabia; taalrebdi@pnu.edu.sa (T.A.A.); fhalkallas@pnu.edu.sa (F.H.A.); 2 Department of Chemistry, Faculty of Science, Cairo University, Cairo 12613, Egypt; ahoda@sci.cu.edu.eg; 3 Chemistry Department, College of Sciences, Taibah University, Yanbu 30799, Saudi Arabia; srfaay@taibahu.edu.sa; 4 Department of Chemistry, Faculty of Applied Science, Umm Al-Qura University, Makkah 24230, Saudi Arabia; rapasha@uqu.edu.sa; 5 Physics Department, Faculty of Science, Taibah University, Yanbu 30799, Saudi Arabia; esobhe@taibahu.edu.sa; 6 Spectroscopy Department, Physics Division Institute, National Research Centre, 33 El Bohouth St. (Former El Tahrir St.), Dokki, Giza 12622, Egypt; 7 Laser Technology Unit, Center of Excellent for Advanced Science, National Research Centre, 33 El Bohouth St. (Former El Tahrir St.), Dokki, Giza 12622, Egypt; emanmwafynrc@gmail.com; 8 Physical Chemistry Department, Advanced Materials Technology and Mineral Resources Research Institute, National Research Centre, Giza 12622, Egypt

**Keywords:** PLD, thin film, ZnO, nanocomposite, catalytic degradation, membrane

## Abstract

Zinc oxide thin film (ZnO thin film) and a silver-doped zinc oxide nanocomposite thin film (Ag/ZnO thin film) were prepared by the technique of the pulsed laser deposition at 600 °C to be applicable as a portable catalytic material for the removal of 4-nitrophenol. The nanocomposite was prepared by making the deposition of the two targets (Zn and Ag), and it was analyzed by different techniques. According to the XRD pattern, the hexagonal wurtzite crystalline form of Ag-doped ZnO NPs suggested that the samples were polycrystalline. Additionally, the shifting of the diffraction peaks to the higher angles, which denotes that doping reduces the crystallite size, illustrated the typical effect of the dopant Ag nanostructure on the ZnO thin film, which has an ionic radius less than the host cation. From SEM images, Ag-doping drastically altered the morphological characteristics and reduced the aggregation. Additionally, its energy band gap decreased when Ag was incorporated. UV spectroscopy was then used to monitor the catalysis process, and Ag/ZnO thin films had a larger first-order rate constant of the catalytic reaction K than that of ZnO thin film. According to the catalytic experiment results, the Ag/ZnO thin film has remarkable potential for use in environmentally-favorable applications.

## 1. Introduction

Thin films are more useful structures to study than their bulk counterparts because of their versatility and positive characteristics. Thin-film assembly of surface-supported metal–metal oxides is becoming more and more ubiquitous, and this is expected to lead to new scientific discoveries as well as expanded application opportunities [1,2,3,4,5]. Examples of applications include membrane-based chemical species separation, silicon integration for electronic detection of volatile compounds, and electrochemical catalysis. ZnO can be considered as a one of the most important materials as it can be used in several application domains. Additionally, ZnO has great piezoelectric properties and exceptional chemical stability due to its impressive semiconductor performance. Over the past few decades, ZnO thin films have been the subject of substantial research [5,6,7,8]. It has a number of clearly stated benefits, including being affordable, plentiful, safe to use, chemically stable, very transparent in the visible and near-infrared spectral area, ecologically benign, and highly catalytic. Since ZnO has a direct bandgap, it offers special features and the versatility to be employed in a variety of applications, including LEDs, gas sensors, optoelectronic components, UV lasers, solar cells, and screen displays [9,10,11,12,13].

The nanocomposite semiconductor thin film is formed by doping the semiconductor thin film with another thin film or metallic nanoparticles, which helps to enhance the optoelectronic properties. These materials provide ZnO with n-type conductivity because the presence of oxygen vacancies leads to the creation of Zn^+2^ interstitials. However, it can be doped to achieve P-type conductivity, making it promising for use in research. According to theoretical research, silver and gold are an appropriate dopant to obtain P-type ZnO. Ag is the most suitable choice among all of the transition metals due to its solubility and the fact that it contains one outermost electron, which accounts for its huge ionic size and orbital energy. In addition, compared to other transition metals, Ag is a cheap, non-toxic, superior electrical and high thermal conductor. The main reason for choosing ZnO doped with Ag is because it improves the characteristics and hinders the trapping of photo-generated electrons, which lengthens the lifespan of charge-separated states [14,15,16,17,18].

The pulsed laser deposition technique (PLD) has been found to be the most efficient and quick method for producing stoichiometric thin film by utilizing multiple targets; these are appealing qualities that make it possible to produce multi-component thin films of high quality. Thin films of metal oxide and other materials related to metal oxide are routinely produced using PLD. Metal nanoparticle-doped oxide films have recently been demonstrated to possess intriguing characteristics such as significant nonlinearity, photocatalysis, etc. [19,20,21,22].

Herein, we synthesized ZnO thin film and developed this structure by embedding the structure with Ag nanostructure to form Ag-doped ZnO nanocomposite thin films by PLD to apply in water treatment. The novelty in this work was based on filling vacancies in the zinc matrix and interstitial zinc atoms with Ag atoms. Different techniques were used to investigate the prepared thin films and determine the changes in their characteristics. Then, the prepared films were used as catalytic-degradable material for the water purification field.

## 2. Experimental Work

### 2.1. Materials

Merck supplied the 4-nitrophenol (4-NP), zinc dust target (Zn), silver target (Ag), sodium borohydride (NaBH_4_), and mono-crystalline quartz substrate (4 × 1 × 0.1 cm^3^), which was washed by chromic acid (purchased from Nasr, Egypt) and then thoroughly rinsed with distilled water, followed by sonication in an ultrasonic bath for 30 min. After treatment, the slides were stored in water until required for use.

### 2.2. Preparation Method

**Figure 1:**
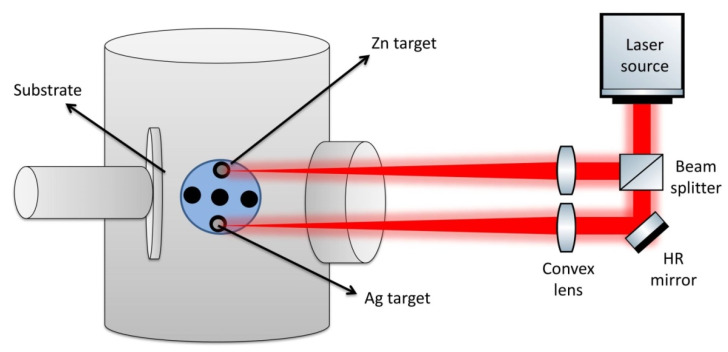
Representative graph of the preparation thin film of Ag/ZnO by PLD.

As previously reported, to adjust the optimum condition of dual-pulsed laser deposition [23,24,25], the thin film was created via PLD of the metal target at the following specifications: 600 °C reaction temperature, 7 ns pulse duration, 1064 nm wavelength, 30 min ablation time, and 7.5 W average power. As shown in Figure 1, pulsed laser ablation was first performed on the Zn to create ZnO thin films on quartz substrate, then on the Ag to create Ag/ZnO nanocomposite thin films on quartz substrate. For Ag-doped ZnO thin film, the film thickness was determined using a profilometer (SurfTestSJ-301) and was found to be 300 nm for ZnO thin film and 500 nm for Ag/ZnO thin film.

### 2.3. Characterization Techniques

UV-visible spectrophotometer (JASCO 570 UV–Vis, Japan), X-ray diffraction (XRD, Schimadzu 7000, Japan), scanning electron microscopic and energy dispersive X-ray (SEM-EDX, Quanta FEG 250, FEI, Czech Republic), and photoluminescence Spectrometer (PL, JASCO, FP-6500, Japan) were used for the characterization.

### 2.4. Catalytic Removal Procedure of 4-NP

The catalytic degradation procedure was conducted by adding the following items together: 100 µL of 0.1 M NaBH_4_ (reducing agent), 2.77 mL of 0.4 mM 4-NP (pollutant) and the prepared thin films were used as a catalytic degradable material. Then, the UV-visible spectrum was examined every 10 min in order to examine the catalytic reaction.

## 3. Results

### 3.1. Investigation of the Prepared Films

**Figure 2:**
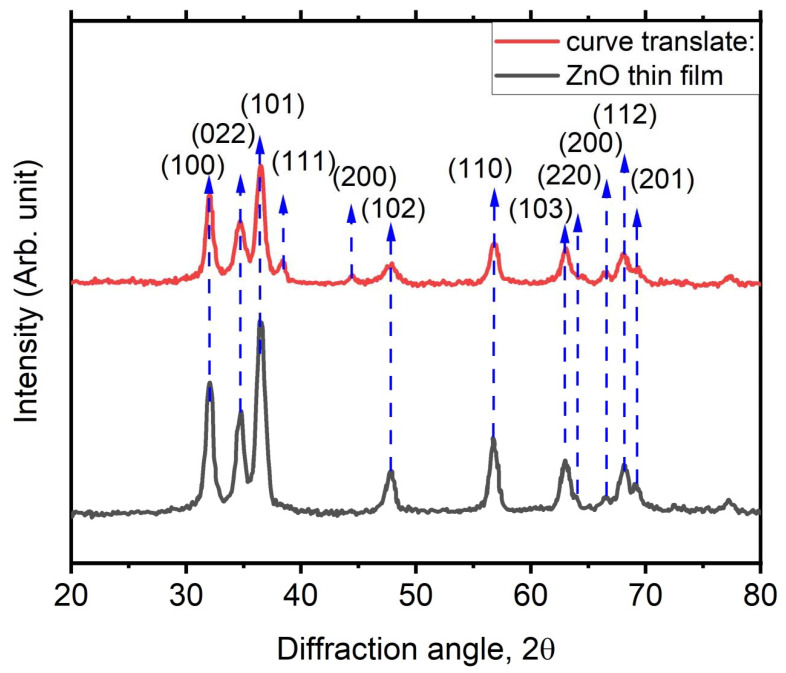
XRD diffractogram of ZnO thin film before and after being embedded with Ag thin films.

Figure 2 shows the diffractogram of ZnO thin film and its embedding with Ag thin films to create a nanocomposite structure thin film. For ZnO thin film, a number of diffraction peaks that appeared corresponded to the reflection planes (1 0 0), (0 2 2), (1 0 2), (1 1 0), (1 0 3), (2 0 0), (1 1 2), and (2 0 1), which is an exact match to the XRD diffraction pattern of pure ZnO (JCPDS card No. 80-0075) [26]. There are no phases that matched any other oxides other than ZnO, which can be seen in the diffractogram for ZnO thin films. Additionally, the diffractogram of the prepared ZnO thin film has a prominent ZnO (0 0 2) peak, indicating that the films are oriented with the c-axis normal to the substrate with a high intensity peak and a sharp shape. In another words, for the ZnO thin film embedded with Ag nanostructure, three XRD peaks of the (1 1 1), (2 0 0), and (2 2 0) planes were indexed to silver, which was fcc phase-matched, confirming Ag as the secondary phase based on the JCPDS card No. 04-0783 [27]. Additionally, the Scherrer formula was used to determine the crystalline size for the (1 0 1) plane, the highest characteristic peak [28,29].

$$D=\frac{\left(0.9\right)\lambda }{\beta cos\theta }$$

where λ is the wavelength of X-ray 1.54 Å, β is FWHM and θ is the diffraction angle of the (1 0 1) plane. The crystallite size decreases from 42 to 19 nm as the Ag dopant concentration increases. As the amount of Ag dopant in thin films increases, the lattice strain between crystallites also increases. More Ag ions cause the lattice to become disordered, which results in a reduction in crystallite size. Because Ag^+^ ions have a higher radius (0.126 nm) than Zn^2+^ ions, the lattice constants rise, increasing the cell volume (0.074 nm).

**Figure 3:**
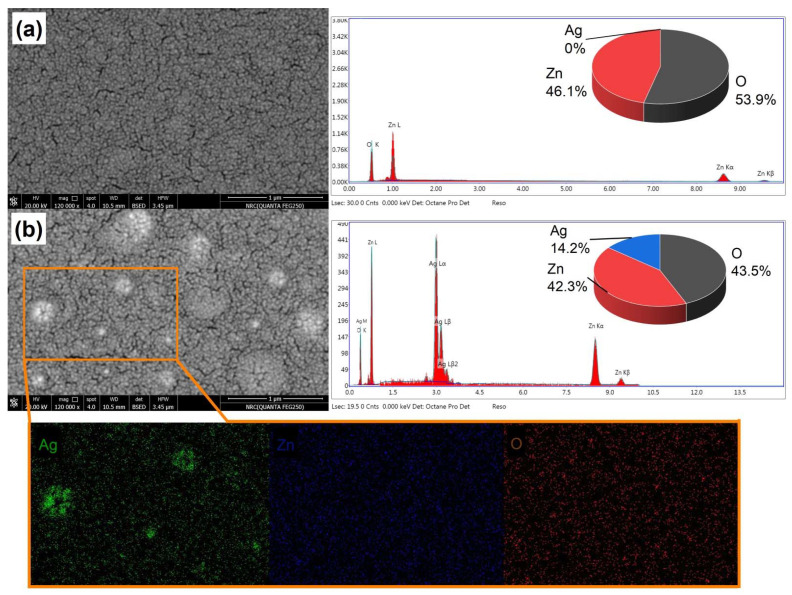
SEM image and elemental analysis of the prepared thin films of (**a**) ZnO and (**b**) Ag/ZnO.

Figure 3 displays SEM images of the annealed ZnO and annealed ZnO/Ag films. The pulsed laser deposition of undoped ZnO films typically results in dense films. It was clear that in the case of Ag-doped films, bright spots started to appear, which is related to the embedding of the ZnO thin film with Ag film. Moreover, the ZnO/Ag thin films’ elemental composition was examined using EDX spectra to establish the presence of Ag. A typical EDX result for thin films is shown in Figure 3. No other components were found, and the top concentrations were Zn, O, and Ag, which was confirmed by elemental mapping.

**Figure 4:**
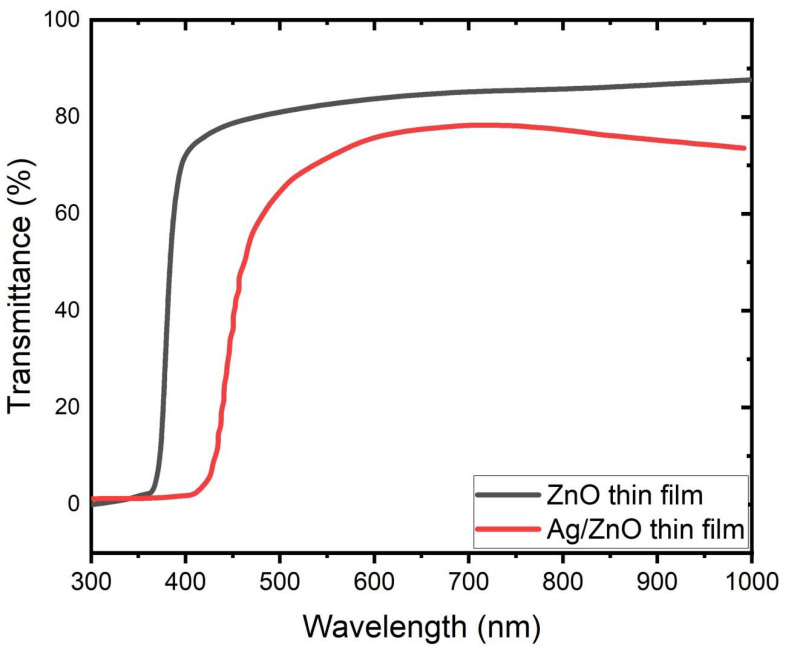
Absorbance spectra of ZnO thin film before and after being embedded with Ag thin films.

UV-vis spectrophotometer was used to evaluate the varying in the optical properties of the prepared strucuture. Figure 4 displays the UV-vis optical absorption spectra of ZnO and Ag/ZnO thin films. Strong excitonic peaks with significant absorbance strength are visible in the sample at 372 nm (pure ZnO). In addition, compared to pure ZnO, the absorption edge shifted to a longer wavelength with Ag deposition to create Ag/ZnO thin films. The inclusion of Ag into the ZnO matrix may be responsible for this shift in the absorption edge. Particles in doped ZnO may be smaller than the exciton Bohr radius, according to a shift in the absorption spectra [30,31,32]. This decrease in the transmittance values of the Ag/ZnO thin film in comparison to ZnO thin film may be caused by grain boundary scattering and the visible light absorption caused by surface plasmon resonance (SPR), which is in excellent agreement with previous studies in the literature [33,34].

**Figure 5:**
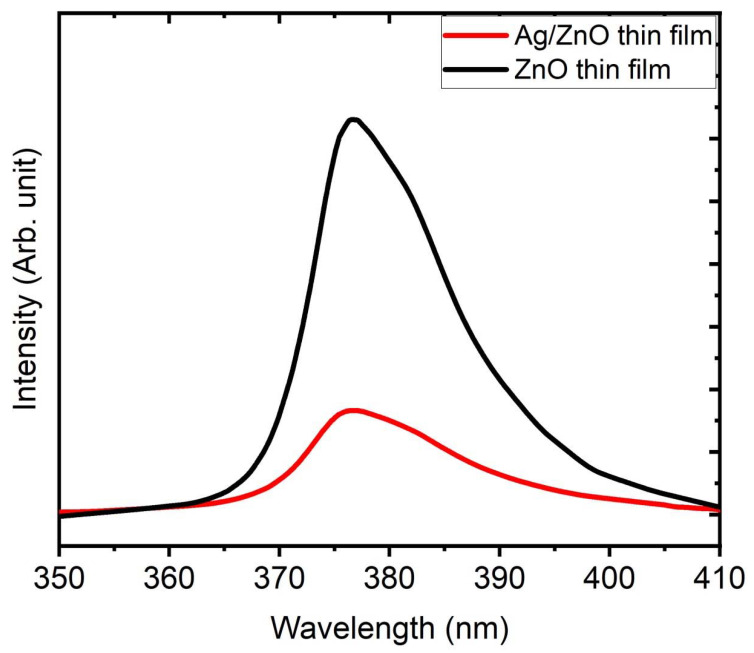
PL spectra of ZnO thin film before and after being embedded with Ag thin films.

Figure 5 displays the PL spectra of thin films, which were calculated using an excitation wavelength of 325 nm and an emission spectrum that ranged from 360 to 410 nm. The samples’ PL spectra show a single violet luminescence peak with a center wavelength of 378 nm. It has been reported that thin ZnO films produced on quartz via PLD emit violet fluorescence, which represents a mixture from the oxygen or zinc vacancies. In other words, in the case of Ag/ZnO nanocomposite thin film, the interface traps between Ag nanoclusters and ZnO grains that exist at the grain boundaries generate violet light from the electronic transition between CB and VB. Additionally, the emission spectra showed a substantial violet emission at 382 nm, which was probably generated by radiate defects brought on by interface traps at the grain boundaries. After Ag was added, this band edge emission peak migrated slightly to a higher value, indicating that the optical bandgap had shrunk. Besides, the presence of oxygen vacancies that interacted with Ag doping also had an impact on the location and intensity of this PL emission. Since Ag^2+^ ions take the position of Zn^+^ ions in the high concentration of Ag-doped ZnO, the peak’s intensity is lower than it is for pure ZnO [35,36,37,38].

### 3.2. Catalytic Activity

**Figure 6:**
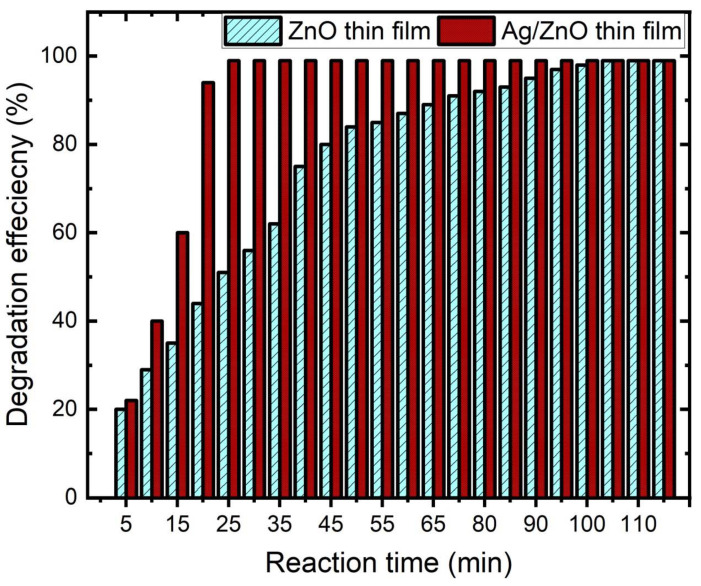
Degradation efficiency of ZnO thin film before and after being embedded with Ag thin films against 4-NP.

Then, using the UV-visible spectra in Figure 6, the catalyst’s catalytic capabilities for reducing 4-NP in an excess of NaBH_4_ were examined. The reduction of 4-NP in the presence of NaBH_4_ at 298 K was catalyzed by the prepared thin films, as seen in the UV spectrum in Figure 6. The absorption of 4-NP was characterized via two peaks (314 nm and 400 nm) while the absorption characteristic peak of 4-NP ion was characterized by one peak (403 nm), and the absorption characteristic peak of the conversion from 4-NP to 4-AP was characterized by one peak (300 nm). This was related to four processes that took place in the catalytic reduction of 4-NP: (1) adsorption of the reactant 4-NP onto the thin ZnO or Ag/ZnO surface; (2) diffusion of 4-NP to the active site; (3) reduction on the site by NaBH_4_; and (4) desorption of the product [39]. According to the UV-vis spectrum, ZnO thin films have a marginal catalytic effect in the first 20 min with an efficiency that does not exceed 35%, whereas Ag/ZnO thin films demonstrate excellent adsorption of 4-NP in the initial period of time with an efficiency that exceeds 90% [40,41,42].

We inserted a single strip of the prepared thin film (1 cm × 2 cm) into a solution of deionized water containing 4-NP and NaBH_4_ at 298 K in order to compare the catalytic effects of the ZnO and Ag/ZnO thin films. Figure 6 shows the degradation efficiency for ZnO thin film and Ag/ZnO thin film against the reaction process. As can be seen, the Ag/ZnO thin film completed the conversion of 4-NP to 4-AP in at least 27 min, whereas the ZnO thin film completed it in 105 min. The catalytic reaction took place after the catalyst accepted the electron donor BH^4−^, transferred it to the electron acceptor 4-NP, and then allowed it to proceed. It might be argued that the rate of decrease was independent of the NaBH4 content because there was an overabundance of NaBH_4_. The solution’s color changed from light yellow-green to dark yellow-green when NaBH_4_ was added to create a 4-NP ion.

**Figure 7:**
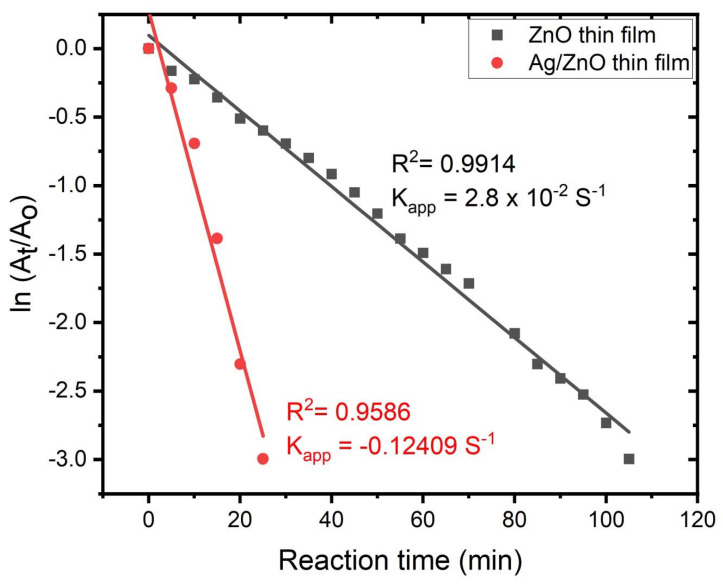
Investigation of ln(A_t_/A_o_) with respect to reaction time (t) for the thin films (ZnO and Ag/ZnO) against 4-NP.

According to Figure 7, which shows the relationship of ln(A_t_/A_o_) versus time (t), the reaction showed a pseudo first-order reaction based on their linear correlation, where A_t_ and A_o_ are the absorbance values at the times (any time, zero time), respectively. The pseudo 1st rate constant of the Ag/ZnO thin film was significantly greater than that of the ZnO thin film. One of the reasons was that the Ag/ZnO structures produced better mass transfer than ZnO structure-assisted catalysts.

## 4. Conclusions

In conclusion, Ag-nanocluster-doped ZnO films were produced at 600 °C by PLD. Several analytical techniques were utilized to examine the produced thin films’ physicochemical characteristics in order to learn more about their optical, structural, and morphological characteristics. These methods revealed the crystalline structure, excellent surface homogeneity, and the production of nano-structural particle size in both the produced films. Then, ZnO structure and the Ag/ZnO structure were investigated for the removal of 4-NP, it was discovered that the degradation efficiency of the Ag/ZnO thin film completed the conversion to 4-AP in at least 27 min, whereas the ZnO thin film competed it in 105 min.
